# Parasitic Infections of Freshwater Fish in Kenya: Prevalence Patterns, Ecological Drivers, and Implications for Aquaculture Sustainability

**DOI:** 10.1155/japr/4352940

**Published:** 2026-04-02

**Authors:** Robert M. Waruiru, Finnan O. Ageng′o, Daniel W. Wanja, Joseph W. Murugami, Kezia W. Maina, Steven K. Mavuti, Patrick W. Kamundia, Charles M. Gichohi, Jacob M. Wainaina, Mercy M. Hamisi, Otieno K. Oginga, Philip N. Nyaga, Paul G. Mbuthia

**Affiliations:** ^1^ Department of Veterinary Pathology, Microbiology and Parasitology, Faculty of Veterinary Medicine, University of Nairobi, Nairobi, Kenya, uonbi.ac.ke; ^2^ Kenya Fisheries Service, Nakuru County, Kenya; ^3^ Department of Veterinary Pathology, Microbiology and Parasitology, Faculty of Veterinary Medicine and Surgery, Egerton University, Egerton, Kenya, egerton.ac.ke; ^4^ Directorate of Veterinary Services, Nairobi, Kenya; ^5^ Directorate of Veterinary Services, Kiambu County, Kenya; ^6^ Pwani University, Kilifi, Kenya, pu.ac.ke; ^7^ Department of Animal Health and Production, School of Natural Resources, Environmental Studies and Agriculture, Maasai Mara University, Narok, Kenya, mmarau.ac.ke; ^8^ Ichthyology Section, National Museums of Kenya, Nairobi, Kenya, museums.or.ke

**Keywords:** digenean trematodes, fish parasites, freshwater aquaculture, Kenya, *Oreochromis niloticus*, risk factors, water quality

## Abstract

Fish parasitic infections present a growing threat to freshwater aquaculture in Kenya, affecting fish health, reducing productivity, and limiting the economic potential of small‐scale and commercial farms. This review provides the first consolidated national checklist of freshwater fish parasites in Kenya, synthesizing evidence from lakes, rivers, and aquaculture systems across multiple counties. By integrating data from diverse host species and production environments, it establishes a comprehensive baseline on parasite diversity, host range, and geographic distribution that has previously been fragmented across isolated studies. Studies were identified through a structured search of scientific databases. The review highlights the widespread occurrence of parasitic infections in economically important species, such as *Oreochromis*, *Clarias*, *Lates*, *Haplochromis*, *Barbus*, and *Cyprinus*. Parasites identified included ectoparasites, a haemoparasite, helminths, and an acanthocephalan. Helminths, especially digenean trematodes, were the most commonly reported. Infection prevalence varied across production systems, with higher diversity and burden in earthen ponds and natural lakes compared with lined or concrete ponds. The most frequently encountered genera were *Gyrodactylus*, *Diplostomum*, *Contracaecum*, and *Proteocephalus*, affecting both wild and cultured fish populations. Risk factors influencing parasite prevalence were linked to environmental conditions and farm management practices. Poor water quality, overstocking, reuse of contaminated nets, retention of pond bottom sediments after harvest, and use of untreated livestock manure were frequently associated with increased infections. Ecological factors such as the presence of wild birds and aquatic snails were also implicated in sustaining transmission cycles. Seasonal flooding further contributed to parasite dispersal in some regions. The review underscores the need for integrated parasite management strategies. This includes routine monitoring using advanced diagnostics, improved biosecurity practices at farm level, targeted training of farmers and extension workers, and reduced dependence on high‐risk inputs such as raw manure. Public awareness on zoonotic risks of fish‐borne parasites should also be strengthened. Investing in research to explore vaccine development and the role of environmental drivers in parasite ecology will be essential in safeguarding the sustainability of Kenya′s freshwater aquaculture sector.

## 1. Introduction

Fish are among the most valuable aquatic resources globally, providing high‐quality protein, essential micronutrients, and polyunsaturated fatty acids that are crucial for human health. In Kenya, however, a significant gap persists between fish demand and supply. Despite the nutritional importance of fish, per capita consumption remains low, averaging less than 5 kg per person per year, far below the recommended 20 kg [1, 2]. As of 2021, Kenya′s total fish production was approximately 146,543 t. Freshwater systems such as lakes, rivers, and aquaculture farms produced 120,873 t, representing the majority of the output. Marine fisheries and mariculture contributed 25,670 t. Notably, aquaculture produced 18,532 t, which remains far below the estimated national potential of 150,000–300,000 t [2]. To meet the rising demand driven by Kenya′s growing population, estimated at over 52 million in 2019 and projected to exceed 57 million by 2030 [3], the Vision 2030 development agenda positions aquaculture as a strategic sector for enhancing food security, reducing poverty, and generating employment.

Nonetheless, aquaculture growth has been constrained by a range of challenges. These include the high cost and limited availability of quality fish feed, lack of certified seed, unreliable water sources, predation, inadequate market access, and disease outbreaks [1, 2, 4–6]. Although intensification is often proposed as a means to boost production, it also increases the risk of disease emergence and transmission, particularly in systems with poor management and weak biosecurity.

Disease remains a significant bottleneck in fish farming across Kenya. Many farmers lack adequate knowledge of fish health, disease prevention, and the importance of biosecurity. Consequently, pathogens such as bacteria, fungi, viruses, and parasites thrive in aquaculture environments, especially under suboptimal conditions [6–8]. These pathogens are often already present in pond water, and outbreaks are typically triggered by environmental stressors, poor water quality, and inappropriate husbandry practices [6, 9, 10].

In recent years, several pathogens have been identified in Kenya′s aquaculture systems. For example, viruses responsible for infectious pancreatic necrosis and infectious hematopoietic necrosis have been isolated from both symptomatic and asymptomatic rainbow trout (*Oncorhynchus mykiss*) in Nyeri County [11, 12]. Concurrent infections involving the digenean *Neascus* sp. (causing black spot disease) and multiple bacterial species have been documented in *Oreochromis niloticus* (Nile tilapia) in Kirinyaga County [13]. Additionally, potentially zoonotic and pathogenic bacteria such as *Aeromonas hydrophila*, *A. veronii*, *Escherichia coli*, *Flavobacterium* sp., *Pseudomonas* sp., *Streptococcus* sp., and *Vibrio* sp. have been recovered from both rainbow trout and Nile tilapia in several regions of the country, including Kiambu, Nyeri, Nyandarua, Machakos, and Taita Taveta counties [9, 10, 13, 14].

Parasitic infections are equally prevalent and often underreported, despite their considerable economic impact. Genera such as *Trichodina*, *Epistylis*, *Dactylogyrus*, and *Gyrodactylus*, commonly found in intensively stocked systems, have been associated with mass mortalities [8, 15]. Others, including *Contracaecum*, *Euclinostomum*, and *Clinostomum*, result in aesthetic damage, leading to rejection of infected fish during market inspection [16–19]. Infections by *Diplostomum* sp., a digenean fluke linked to cataracts and impaired vision in fish, have been widely documented and are associated with reduced feeding efficiency and stunted growth [15, 17, 20]. Moreover, endoparasites with zoonotic potential, including *Contracaecum* (nematode), *Euclinostomum* (trematode), and *Acanthocephalus* sp., have been isolated from various aquaculture facilities across the country [8, 18, 21, 22].

Despite numerous localized studies, Kenya lacks a consolidated national synthesis linking fish parasite diversity with aquaculture practices, ecological drivers, and policy‐relevant risk factors. Although several fish‐borne parasites reported from Kenyan freshwater systems are recognized as zoonotic in other regions, there is currently limited direct evidence of human infection attributable to fish consumption in Kenya. Their relevance should therefore be interpreted primarily as a potential public health concern warranting precautionary surveillance rather than as a demonstrated zoonotic burden.

This review synthesises existing studies to produce the first consolidated national checklist of fish parasites in Kenya, assess their current distribution and status, and examine their implications for aquaculture productivity. In doing so, it identifies critical knowledge gaps, particularly in diagnostic capacity and the development of targeted, evidence‐based control measures. Overall, the review highlights the need to strengthen parasite management strategies to support sustainable aquaculture development, fish health, and national food security.

## 2. Methodological Approach

A targeted narrative review approach [23] was employed to synthesize evidence on the prevalence and risk factors associated with parasitic infections of freshwater fish in Kenya. The review focused on studies published between 1993 and 2025, a period that captures the expansion of aquaculture and intensified fish health research in the country.

### 2.1. Search Strategy and Scope

Literature searches were conducted across major scientific databases including Scopus (https://www.scopus.com/), Web of Science (https://www.isiknowledge.com/), PubMed (https://pubmed.ncbi.nlm.nih.gov/), Google Scholar (https://scholar.google.com/), ScienceDirect (https://www.sciencedirect.com/), and Semantic Scholar (https://www.semanticscholar.org/), to identify peer‐reviewed journal articles published by higher learning and research institutions in Kenya, reports and policy documents published. The search strategy combined keywords and Boolean operators: “fish parasites” AND “freshwater fish” AND “Kenya” AND (“aquaculture” OR “fish health” OR “fish diseases”).

### 2.2. Inclusion and Exclusion Criteria

An initial screening of approximately 89 records was conducted based on titles and abstracts. Following removal of duplicates and application of eligibility criteria, 37 peer‐reviewed articles and academic theses were included in the final synthesis. Reference lists of eligible articles were also manually screened to identify additional relevant studies.

Inclusion criteria comprised studies reporting on parasite occurrence, prevalence, risk factors, diagnostic approaches, or management implications in freshwater fish (wild or farmed) within Kenya. Exclusion criteria included incomplete reports, studies lacking clear methodological descriptions, and publications where the ecological or geographic context could not be reliably established.

Although the review primarily included studies conducted by Kenyan universities and research institutions, this criterion was applied to ensure local ecological relevance, methodological comparability, and contextual accuracy rather than institutional affiliation per se. This approach allowed for synthesis of data generated under similar environmental, production, and management conditions characteristic of Kenyan freshwater systems.

### 2.3. Data Extraction and Thematic Synthesis

Data were extracted using a standardized matrix capturing authorship, year of publication, study location, host species, parasite taxa, prevalence estimates, identified risk factors, diagnostic methods, and control or management recommendations. Extracted data were subsequently grouped thematically to facilitate synthesis across parasite groups, production systems, and ecological drivers.

### 2.4. Review Credibility and Limitations

Most of the articles were recovered from high‐quality peer‐reviewed journals and some theses obtained from different universities′ repositories supervised by competent researchers. However, the review was not exhaustive in the sense that most of the grey literature was not incorporated.

## 3. Results

### 3.1. Distribution of Fish Samples and Parasites in Kenya

Fish farming in Kenya has experienced continuous growth, particularly in areas with favorable climatic conditions. This growth has been largely driven by the economic stimulus initiatives of the previous government, which heavily invested in fish production through subsidies for certified fingerlings and feeds, pond construction, and employment of extension fisheries officers [24, 25]. However, the potential is yet to be met as farmers experience challenges such as poor seeds, unaffordable and unavailable feeds, and fish parasitism [2, 26].

The University of Nairobi and other universities and research centers in Kenya have been conducting research on fish to investigate fish parasites as a limiting factor to fish production in counties with high potential of aquaculture production in Kenya as shown in Table 1. From studies under review, sampled fish were infected by 45 different parasite genera (Table 1).

**Table 1:** Distribution of parasites by county, fish species, and source reference.

County/lake	No. of fish sampled	Name of fish species sampled	Parasite genera	Citation text
Kirinyaga	820	*O. niloticus* (494)	*Neascus*; *Diplostomum*; *Clinostomum*; *Gyrodactylus*; *Dactylogyrus*; *Acanthocephalus*; *Piscicola*; *Pseudophyllediancestode*; *Contracaeum*; *Proteocephalus*	Wanja et al. [20]; Murugami et al. [17]; Waruiru et al. [19]; Murugami et al. [27]; Gichohi et al. [22]; Shigoley et al. [28]
		*C. gariepinus* (274)	*Diplostomum*; *Paracamallanus*; *Contracaecaum*; *Dactylogyrus*; *Gyrodactylus*; *Acanthocephalus*; *Pseudophylledian*; *Proteocephalus*; *Caryophyllaidea*	Waruiru et al. [19]; Murugami et al. [17]; Murugami et al. [27]; Gichohi et al. [22]
		Ornamental fishes (52)	*Dactylogyrus*; *Acanthocephalus*; *Contracaecaum*;	Waruiru et al. [19]
Homa Bay	162	*O. niloticus* (150)	*Trypanosoma*; *Epistylis*; *Gyrodactylus*; *Dactylogyrus*; *Trichodina*; *Argulus*	Kamundia et al. [29]; Wainaina et al. [30];
		*L. niloticus* (12)	*—*	Kamundia et al. [29]
Kiambu	260	*O. niloticus* (260)	*Diplostomum*; *Clinostomum*; *Gyrodactylus*; *Dactylogyrus*; *Acanthocephalus*	Maina et al. [17]
Kericho	90	*O. niloticus* (90)	*Dactylogyrus*; *Diplostomum*; *Epistylis*; *Trichodina*; *Riboscyphidia*; *Paracamallanus*; *Camallanus*; *Contracaecum*	Ageng′o et al. [8]; Ageng′o et al. [21]
Nakuru	370	*O. niloticus* (370)	*Diplostomum*; *Acanthocephalus*; *Euclinostomum*; *Trichodina*; *Contracaecum*; *Camallanus*; *Ichthyobodo*; *Microsporidia*; *Cryptobia*; *Chilodonella*; *Cichlidogyrus halli*; *Cichlidogyrus sclerosus*; *Gyrodactylus*; *Tylodelphys*; *Heterophyes*; *Amirthalingamia macracantha*; *Argulus*; *Lamproglena monodi*; *Lernaea cyprinacea*	Ageng′o et al. [21]; Ojwala et al. [31]
Lake Naivasha 1520		*O. leucostictus* (792)	*Trichodina*; *Trichodinella*; *Myxobolus*; *Epistylis*; *Cichlidogyrus*; *A. macracantha*; *Contracaecum multipapillatum*; *Heterophyes*; *Tylodelphys*; *C. sclerosus*; *C. tilapiae*; *C.halli*; *S. gravivaginus*; *Polyacanthorhynchus kenyensis*; *Cyclustera*; *Clinostomum*	Otachi et al. [32]; Otachi et al. [33]; Rindoria et al. [34]; Aloo, [35]
		*T. zillii* (466)	*Trichodina*; *Trichodinella*; *Cichlidogyrus*; *A. macracantha*; *C. multipapillatum*; *Heterophyes*; *Tylodelphys*; *P. kenyensis*; *Cyclustera*; *Clinostomum*	Otachi et al. [32]; Aloo, [35]
		*C. carpio* (145)	*Trichodina*; *Trichodinella*; *Chilodonella*; *Tetrahymena pyriformis*; *Dactylogyrus minutus*; *Dactylogyrus extensus*; *Acolpenteron*; *Tylodelphys*	Otachi et al. [32]
		*B. paludinosus* (67)	*Trichodina*; *Trichodinella*; *Ichthyophthirius multifiliis*; *T. pyriformis*; *Cryptobia*; *Epistylis*; *Dactylogyrus*; *A. macracantha*; *Contracaecum*; *Ligula intestinalis*	Otachi et al. [32]; Britton et al. [36]
		*O. niloticus* (50)	*C. sclerosus*; *C. tilapiae*	Rindoria et al. [34]
Bomet	60	*O. niloticus* (60)	*Dactylogyrus*; *Epistylis*; *Trichodina*; *Riboscyphidia*; *Paracamallanus*; *Camallanus*	Ageng′o et al. [8]; Ageng′o et al. [21]
Lake Baringo	573	*Barbus lineomaculatus* (20)	*L. intestinalis*	Britton et al. [36]
		*O. niloticus* (553)	*Argulus*; *A. macracantha*; *Apharyngostrigea*; *Neascus*; *C. sclerosus*; *Clinostomum*; *Contracaecum*; *Euclinostomum*; *Heterophyes*; *Microsporidia*; *Trichodina*; *Tylodelphys*	Adamba et al. [37]; Chebon et al. [38]
Chelaba Spring	126	*O. niloticus* (126)	*C. sclerosus*; *Clinostomum*	Adamba et al. [37]
Lake Bogoria	115	*O. nilocus* (115)	*C. sclerosus*; *Clinostomum*; *Contracaecum*; *Heterophyes*	Adamba et al. [37]
Lake Victoria	412	*L. niloticus* (103)	*Henneguya ghaffari*; *Diplectanum lacustris*; *C. multipapillatum*; *Cucullanus*	Outa et al. [39]
		*O. niloticus* (165)	*Myxobolus*; *C. sclerosus*; *C. halli*; *Cichlidogyrus tilapiae*; *Cichlidogyrus quaestio*; *Scutogyrus longicornis*; *Gyrodactylus cichlidarum*; *Gyrodactylus malalai*; *A. macracantha*; *Tylodelphys*; *Neascus*; *Diplostomum*; *Clinostomum tilapiae*; *Euclinostomum heterostomum*; *C. multipapillatum*; *Acanthogyrus*	Outa et al. [39]
		*Haplochromis piceatus* (82)	*Myxobolus*; *Cichlidogyrus gillardinae*; *A. macracantha*; *Tylodelphys*; *Neascus*; *Diplostomum*; *E. heterostomum*; *C. multipapillatum*; *Acanthogyrus*	Outa et al. [39]
		*H. humilior* (62)	*Myxobolus*; *C. gillardinae*; *A. macracantha*; *Tylodelphys*; *Neascus*; *Diplostomum*; *E. heterostomum*; *C. multipapillatum*; *Acanthogyrus*	Outa et al. [39]
Uasin Gishu	195	*O. niloticus* (195)	*Diplostomum* sp.	Migiro et al. [40]
Taita Taveta	111	*O. niloticus* (34); *O. jipe* (66); Hybrid (*O. nilotus* X *O. jipe*) (11)	*Dactylogyrus*; *Diplostomum*; *Acanthocephalus*; *Gyrodactylus*; *Euclinostomum*; *Camallanus*; *Contracaecum*	Ageng′o et al. [41]
Bungoma	394	*O. niloticus* (394)	*Acanthocephalus*; *Procamallanus*; *Philometroides*	Mukwabi et al. [42]
Nyeri	366	*O. niloticus* (277); *C. gariepinus* (89)	*Diplostomum*; *Clinostomum*; *Gyrodactylus*; *Dactylogyrus*; *Paracamallanus*; *Contracaecasm*; *Acanthocephalus*; *Trichodina*;	Mavuti et al. [15]

Abbreviations: *B*, *Barbus*; *C. carpio*, *Cyprinus carpio*; *C. gariepinus*, *Clarias gariepinus*; *H*, *Haplochromis*; *L. niloticus*, *Lates niloticus*; *O. jipe*, *Oreochromis jipe*; *O. niloticus*, *Oreochromis niloticus*.

*Oreochromis* sp. was the most infected fish with 37 genera, followed by *Clarias* sp. 11, *Barbus* sp. 10, *Haplochromis* sp. 8, *Cyprinus carpio* 6, *Lates niloticus* 4, and ornamentals with 3 genera. These parasites were grouped into 6 nematodes, 7 digeneans, 6 monogeneans, 5 crustaceans, 3 acanthocephalans, 6 cestodes, and 12 protozoans. Nematodes recovered included *Contracaecum*, *Paracamallanus*, *Cucullanus*, *Procamallanus*, and *Philometroides* species. The digeneans identified were *Diplostomum*, *Neascus*, *Clinostomum*, *Euclinostomum*, *Heterophyes*, and *Apharyngostrigea* species. Monogeneans comprised *Gyrodactylus*, *Dactylogyrus*, *Cichlidogyrus*, *Acolpenteron*, *Diplectanum lacustris*, and *S. gravivaginus*. The protozoans recovered included *Trypanosoma*, *Epistylis*, *Trichodina*, *Ichthyobodo*, *Microsporidia*, *Cryptobia*, *Chilodonella*, *Myxobolus*, *Tetrahymena pyriformis*, *Ichthyophthirius multifiliis*, and *Henneguya ghaffari*. Cestodes identified were *Pseudophyllidean* cestodes, *Proteocephalus*, *Caryophyllaeidae*, *Amirthalingamia macracantha*, *Cyclustera*, and *Ligula intestinalis*. The acanthocephalans recovered were *Acanthocephalus*, *Polyacanthorhynchus kenyensis*, and *Acanthogyrus*. Three out of four genera infecting *L. niloticus*, such as *H. ghaffari*, *D. lacustris*, and *Cucullanus*, did not infect other fish species.

### 3.2. Parasitic Infections in the Lakes and Farms

When comparing parasites in fresh waters, there was slight difference in parasite diversity with lakes being infected with 30 genera whereas farms had 29. Four fish species from the wild were reviewed because of their economic value, *Barbus* sp. *Haplochormis* sp., *C. carpio*, and *Late niloticus.* Parasitic richness varied across the examined fish taxa, with *L. niloticus* being the most depauperate in parasite as recorded in Lake Victoria [39]. Outa et al. [39] recorded *H. ghaffari*, *D. lacustris*, *Contracaecum* sp., and *Cucullanus* sp. Three genera were not isolated from other fishes both in lakes and farms except *Contracaecum* sp. These three species tend to specific *L. niloticus*. Abdel‐Baki et al. [43] isolated *H. ghaffari* infecting *L. niloticus* in River Nile, and Hamed Hamouda et al. [44] also isolated *D. lacustris* in Lake Nasser in Egypt. While *Cucullanus* sp. infect wide range of fish mostly *Anguilla sp.* [45]. Kamundia et al. [29] conducted a study in Lake Victoria and investigated the existence of *Trypanosoma* sp., a haemoparasite in *O. niloticus* and *L. niloticus. L. niloticus* recorded no parasites and this may be due to the biology of the fish where it inhabits deep oxygenated waters; hence, water quality parameters are good for fish health [46]. However, the small number of *L. niloticus* examined may have influenced the findings.

There are few studies on *Haplochromis* sp. parasites in Kenya despite playing a major role in food security to the local population. Outa et al. [39] recorded the first parasites that included *Myxobolus*, *Cichlidogyrus gillardinae*, *A. macracantha*, *Tylodelphys*, *Neascus*, *Diplostomum*, *Euclinostomum heterostomum*, *Contracaecum multipapillatum*, and *Acanthogyrus*. Parasites from *Haplochromis* sp. have also been recovered in species such as *Oreochromis* sp., *Tilapia zillii*, *Barbus* sp., and *C. carpio* in Kenya [31, 38, 41, 47]. *C. carpio* is an invasive fish in Lake Naivasha, and the study shows the dominant parasites being monogeneans such as *Dactylogyrus minutus* and *D. extensus*, introduced together in the Lake with *C. carpio*, hence species‐specific [32].

In aquaculture, widespread infection of dominant farmed fish such as *Oreochromis* sp. and *C. gariepinus* by different parasite genera was mainly due to intensification of inland‐based culture systems like earthen, liner and concrete ponds, and plastic tanks [2].

Intensification of fish farming leads to deterioration of water quality parameters, which may be due to buildup of metabolic waste or accumulation of excessive uneaten feed within the culture systems. This disturbs the state of equilibrium of food and parasites leading to increased infections [48, 49]. Bad aquaculture practices like sharing of nets without treatment among farmers, overstocking, leaving pond bottom sediments after harvesting and restocking ponds without drying, or leaving fallow for the required period and cleaning also increased fish parasite prevalence in different culture systems [8, 15, 17, 21].

## 4. Importance of Ectoparasites

Among ectoparasites reported in Kenyan aquaculture systems, *Dactylogyrus* and *Gyrodactylus* species were the most prevalent monogeneans affecting farmed fish [15, 18, 21, 30]. *Dactylogyrus* sp. primarily infects the gills of *O. niloticus* and *C. gariepinus* and is morphologically characterized by a scalloped head with an anterior eye spot [50], making them distinguishable during microscopic diagnosis.

**Figure 1:**
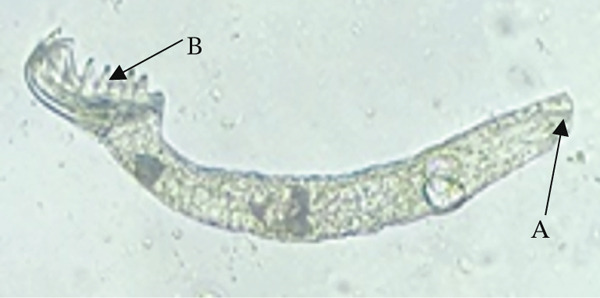
*Gyrodactylus* sp. anterior end showing a V‐shaped head (Arrow A) and posterior end having opisthohaptor (Arrow B) recovered from the skin of Nile tilapia in Kericho County, Kenya (source: Ageng′o et al. [21]).

*Gyrodactylus* sp. (Figure 1), on the other hand, tends to colonise the skin and fins of infected fish. *Gyrodactylus* sp. is viviparous, and it is common to observe a developing embryo within an adult parasite, which itself may contain a smaller, second‐generation embryo [15]. They are identified by their V‐shaped head, which lacks eye spots, and a posterior opisthohaptor used for attachment [15, 50]. In a study done in commercial tilapia hatcheries around Lake Victoria in Homabay County, only *Gyrodactylus* sp. was recovered. High prevalence of this parasite was in larvae, fries, and fingerlings due to their high buildup in numbers. *Gyrodactylus* sp. spread faster and increased in numbers where stocking density is high [51]. This parasite has been reported in different parts of the world to cause mass mortalities of farmed fish, and in hatcheries, it can be catastrophic as the fish are still in developmental stages [30, 52]. Sharing of hatchery equipment such as scoop nets, egg collectors, mugs, and fin meshed from different incubators or fish holding systems contributed to the spread of other ectoparasites such as *Dactylogyrus*, *Epistylis*, and *Trichodina* species in commercial hatcheries [30]. There are numerous species of *Gyrodactylus* with varying pathogenicity; the presence of *Gyrodactylus salaris*, a related species responsible for gyrodactylosis in salmonids, is of international concern and has been listed by the World Organization for Animal Health (WOAH) as a notifiable aquatic disease [51]. Although *G. salaris* has not been confirmed in Kenya, the genus remains highly relevant due to its potential for rapid transmission in high‐density farming systems.

Ectoparasites, particularly monogeneans (*Gyrodactylus*, *Cichlydogyrus*, and *Dactylogyrus*) play a significant role in fish health challenges within Kenyan aquaculture. Several studies have reported higher prevalence rates of these parasites in earthen ponds than in other culture systems [15, 17, 21]. This pattern is largely attributed to the way these ponds are constructed, typically in wetland areas that are continuously fed by streams, rivers, or groundwater. These water sources often carry parasite eggs and free‐swimming infective larvae, increasing the likelihood of transmission [15]. Interestingly, although studies in Kenya show prevalence to be higher in earthen ponds, the intensity of monogenean infections tends to be greater in liner ponds. This is likely due to the stagnant nature of water in these systems and the buildup of organic matter, which creates an ideal environment for parasite proliferation [15, 21].

Globally, *Gyrodactylus* sp. has been linked to significant fish mortality events, posing a major threat to the sustainability of aquaculture operations [51, 52]. Monogeneans attach to the skin, fins, and gills of their hosts, often causing open wounds and damaging the fish′s protective epidermis. This not only compromises fish health directly but also increases vulnerability to secondary infections [53].

Despite frequent detection of *Gyrodactylus* spp., species‐level identification remains rare, limiting risk assessment for notifiable species.

**Figure 2:**
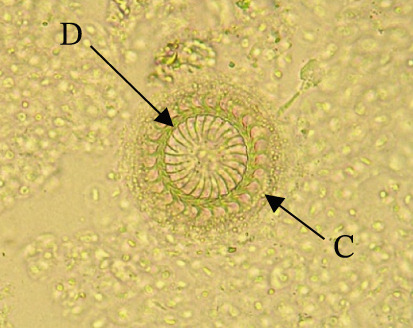
Round‐shaped *Trichodina* sp. from a catfish from Nyeri County, Kenya showing denticle (D) and cilia (C) (source: Mavuti et al. [15]).

Among the most common ectoparasites reported in Kenyan aquaculture was *Trichodina* sp. (Figure 2). Recognized by its round dorsal shape and ring of hook‐like denticles, *Trichodina* sp. moves rapidly using cilia, and its adhesion and movement cause abrasions on host tissues primarily through the action of its denticles and hooks. At high intensities, these lesions contribute to significant mortality, especially in fry, and often serve as gateways for secondary bacterial infections [50, 53]. Otachi et al. [33] recovered *Acolpenteron* sp., *D. minutus*, and *D. extensus* in *C. carpio* from Lake Naivasha. In a study, Wainaina et al. [30] examined *Trichodina* prevalence across developmental stages in hatcheries and found no infections in eggs or larvae. Prevalence rose to 20% in fry, 18% in fingerlings, and 13% in broodstock. This gradient was linked to feeding regimes; fry receive higher protein feeds that may contribute to excess organic waste and nutrient buildup, creating favorable conditions for parasite proliferation.

**Figure 3:**
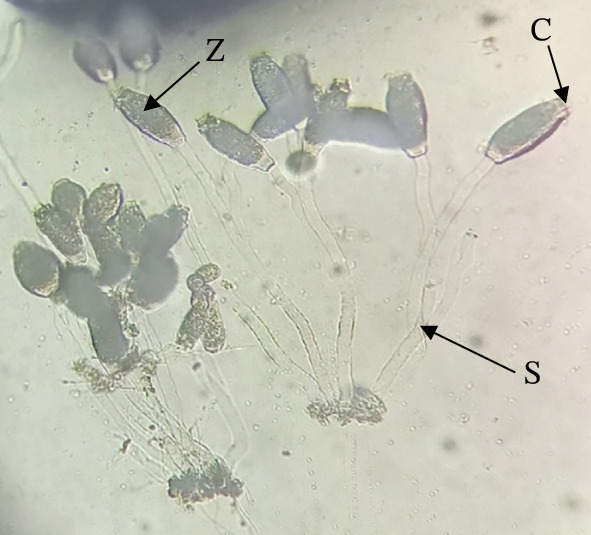
*Epistylis* sp. recovered from the skin of *Oreochromis niloticus* showing zooid forms (Z), stalk (S), and cilia (C) (source: Ageng′o et al. [21]).

Ectoparasitic protozoa, especially *Epistylis* and *Riboscyphidia* species, were common in Kenyan freshwater aquaculture, particularly on the skin and external surfaces of *O. niloticus* [21, 30]. These parasites tend to be more prevalent and intense in broodstock than in fingerlings, likely due to differences in rearing environments. Fingerlings are typically raised in hatcheries under controlled, hygienic conditions, whereas brooders are often maintained in more open, less regulated systems [30]. These parasites anchor onto fish surfaces to feed, and although they may seem harmless at first, their effects are far from superficial. *Epistylis* sp. (Figure 3), for example, causes visible white patches on fins, jaws, and gill covers, damaging the fish′s mucosal barrier and leaving them vulnerable to secondary bacterial infections mainly caused by *A*. *hydrophila* [50, 53].

**Figure 4:**
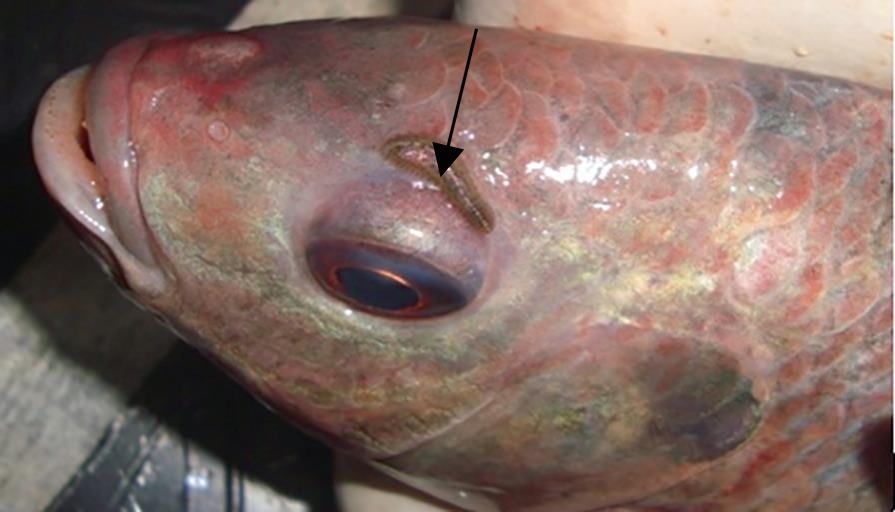
*Piscicola* sp. (arrow) attached to *Oreochromis niloticus* from Kirinyaga County, Kenya (source: Waruiru et al. [19]).

Ectoparasites play a critical role in freshwater fish health, particularly in systems under aquaculture or environmental stress. Among the most notable is *Piscicola* sp. (Figure 4), a freshwater leech from the phylum Annelida, commonly found in lakes, ponds, and streams. These leeches use their well‐developed oral and caudal suckers to attach to fish skin, feeding on blood and tissue fluids.

Although small infections are often tolerated by the host, high parasite loads can result in significant pathology. Reported impacts include skin ulceration, hemorrhaging, necrosis, and anemia, mainly due to the cumulative tissue damage at attachment sites. In addition, these lesions may act as entry points for secondary infections from bacteria and fungi, compounding health risks [50, 53].

Beyond direct damage, leeches are also important from an epidemiological perspective. They are known vectors of trypanosomes, which affect both marine and freshwater fish species [54]. This dual role, as both parasite and pathogen vector, underscores their importance in fish health management and surveillance programs.

## 5. Importance of Endoparasites

### 5.1. Haemoparasites

**Figure 5:**
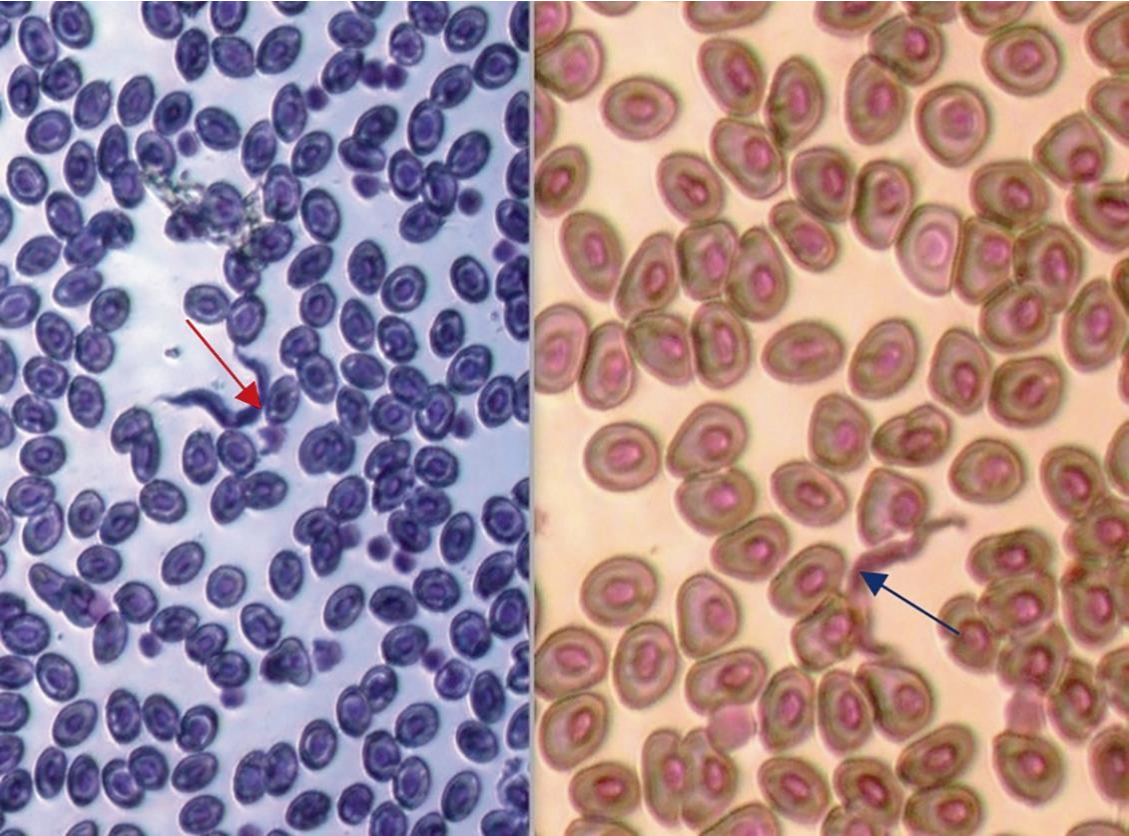
*Trypanosoma* sp. with a pale staining eosinophilic flagellum, pointed anterior end (red arrow), and a posterior end (blue arrow) in Giemsa‐stained blood smears from *Oreochromis niloticus* (source: Kamundia et al. [29]).

Trypanosomes are protozoan haemoflagellates infecting vertebrate species and may cause death in infected hosts including fish and humans by feeding on nutrients in the blood and causing agglutination of red blood cells or proliferation of lymphatic organs or kidney immunopathology [55]. With the global development of high‐density farming in marine and fresh fish aquaculture systems, severe disease or death due to trypanosomiasis has been reported [55, 56]. In Kenya, Kamundia et al. [29] carried out a study on parasites infecting fish in Homa Bay County, along the shores of Lake Victoria. *Trypanosoma* sp. were found in blood smears of *O. niloticus* (Table 2; Figure 5).

**Table 2:** Prevalence and intensity of trypanosomes in *Oreochromis niloticus* and *Lates niloticus.*

Fish species	Sample size (*N*)	Number of fish species infected (*n*)	Number of parasites isolated	Prevalence (%)	Mean intensity
*Oreochromis niloticus*	10	5	7	50	1.4
*Lates niloticus*	12	0	0	0	0

*Note:* Source: Kamundia et al. [29].

No research has found *L*. *niloticus* to be infected with *Trypanosoma* sp. [57] and agreed with the finding of Kamundia et al. [29]. The parasite is spread to fish by leeches that reside in shallow areas of the lake hiding among aquatic flora or attached to organic debris; hence, the relatively high prevalence of *Trypanosoma* sp. in *O. niloticus* as they feed on zooplankton [4, 58]. Kamundia et al. [29] did not find any trypanosomes in *L. niloticus*, and this was because they occupy a different ecological niche of deep‐water, contrary to leeches [29]. Fish infections by trypanosomes in the majority of cases are asymptomatic. However, when intensity is high in the bloodstream, they can severely affect the health of the fish, leading to anemia and massive reduction of thrombocytes [57]. Effective treatment of fish trypanosomiasis is not yet available due to lack of knowledge of the biology and pathogenesis of these haemoflagellates [55].

### 5.2. Digenean Trematodes

**Figure 6:**
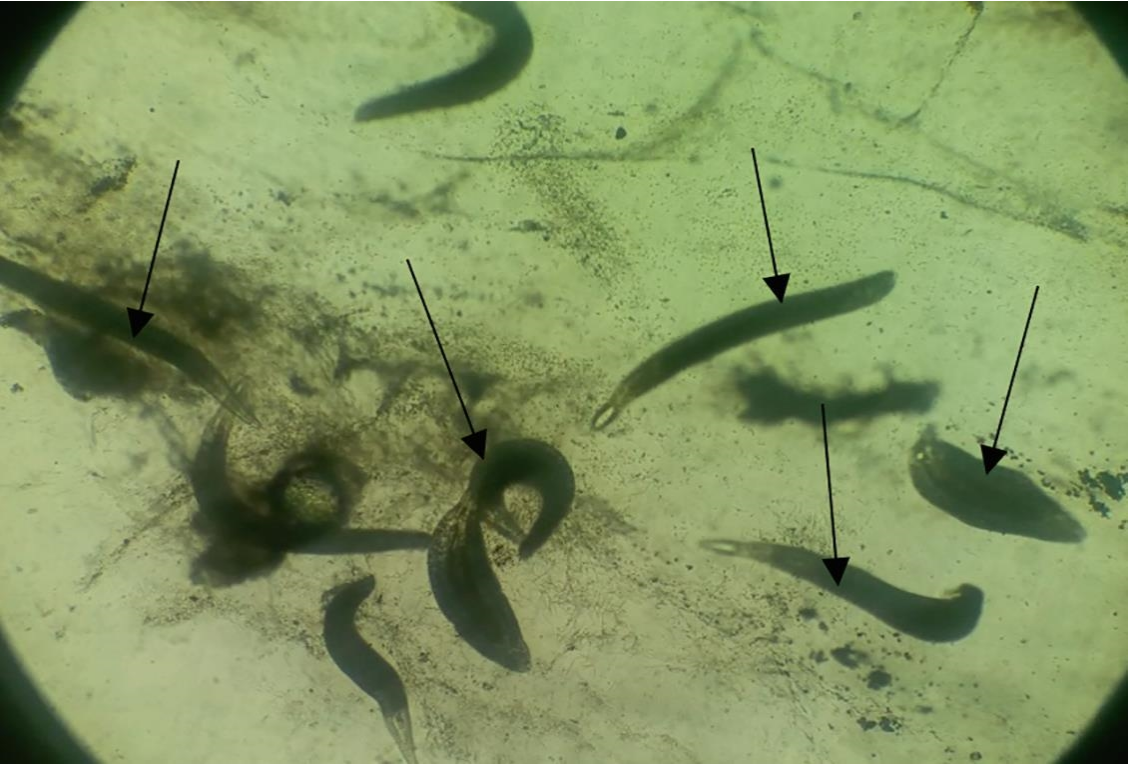
*Diplostomum* sp. (arrows) recovered from vitreous humor of the eyes of *Oreochromis niloticus* from Nyeri County, Kenya (source: Mavuti et al. [15]).

*Diplostomum* sp. metacercariae (Figure 6) were recovered swimming in the vitreous humor of *C. gariepinus* and *O. niloticus* [15, 18, 21, 41]. *Diplostomum* sp. occurrence was higher in earthen ponds compared with other culture systems due to the availability of snails (i.e., *Melanoides* sp.), which are the intermediate hosts [17, 50]. Metacercariae invade the vitreous humor of fish eyes, infect lens and ocular tissues, leading to blindness and cranial deformation [59]. Consequences of blindness include exposure of fish to predation, emaciation, poor growth, and subsequent death [59, 60]. Other parasites from the Diplostomidae family, such as *Apharyngostrigea* sp., have been recovered from the eyes of *O. niloticus* in Lake Baringo [38] and *Tylodelphys* sp. from lakes Baringo, Victoria, and Naivasha, and farms in Nakuru County [31, 32, 39, 47].

*Neascus* sp. metacercariae (Figure 7) were reported infecting the skin, mouth, fins, gills, muscles, and eyes of *O. niloticus* in Kirinyaga County at a low prevalence of 4.1% and high intensity of 190.3 [20]. The parasite usually causes black spot disease, making fish lose aesthetic value, leading to rejections in the market and massive economic losses [61, 62].

Figure 7Metacercariae of *Neascus* sp. (arrows) appearing as black spots in tail fins (a) and gills (b) of *Oreochromis niloticus* from Kirinyaga County, Kenya (source: Wanja et al. [20]).

**Figure:**
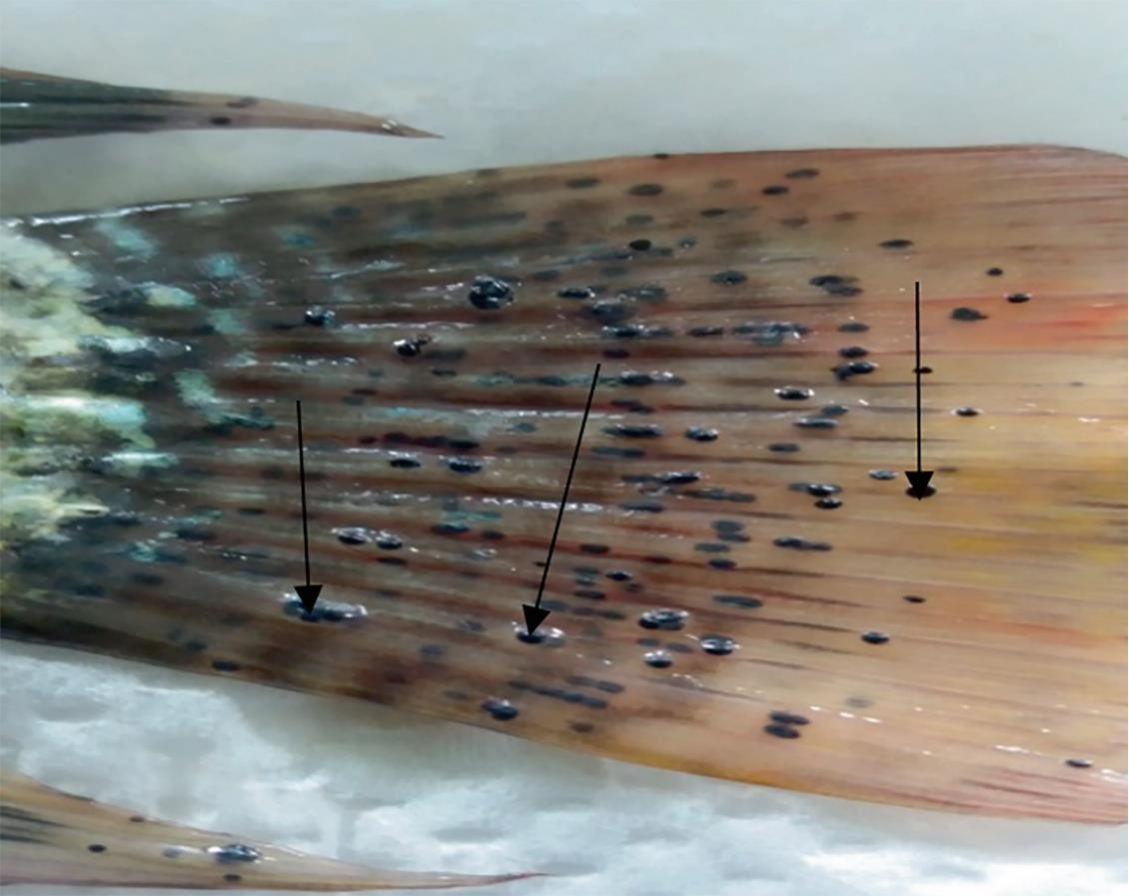
(a)

**Figure:**
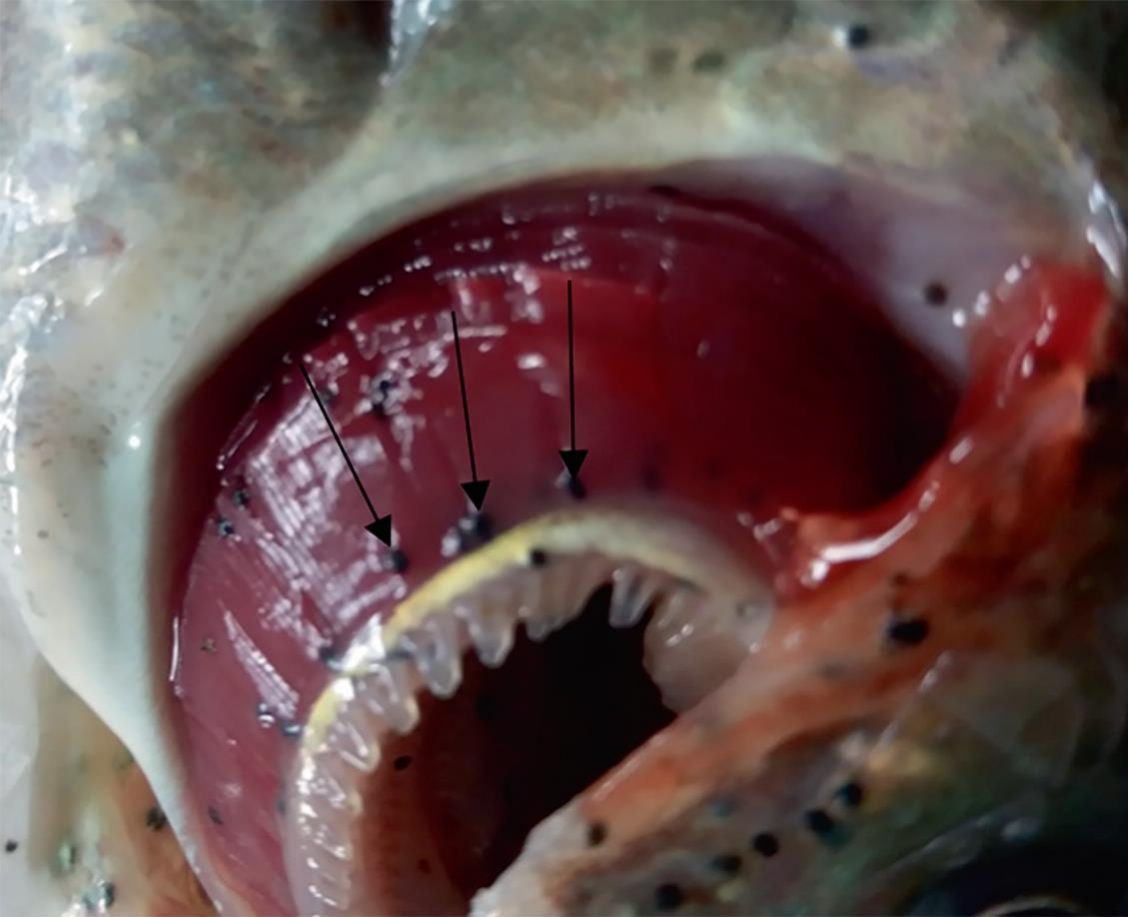
(b)

**Figure 8:**
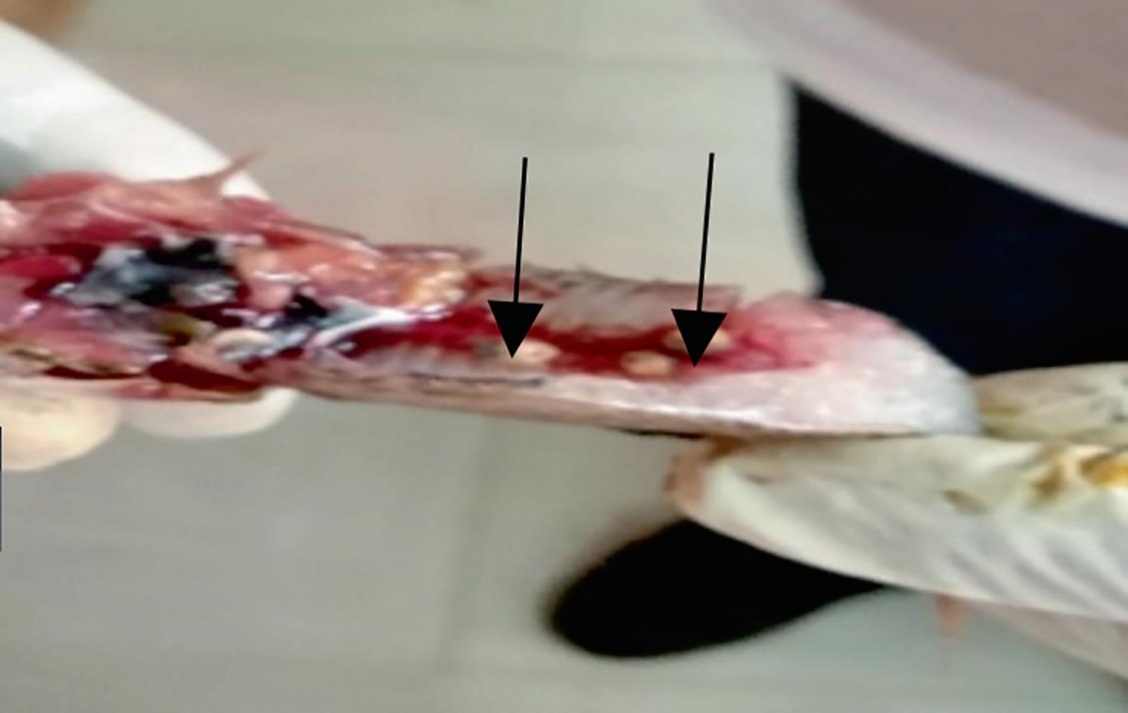
*Euclinostomum* sp. encysted in kidneys (black arrows) of *Oreochromis niloticus* from Nakuru County, Kenya (source: Ageng′o et al. [21]).

Another digenean trematode, *Euclinostomum* sp. (Figure 8) was recovered as round cysts in the kidneys of *O. niloticus* harvested in liner ponds in Nakuru County. In other fish species like guppies *Poecilia reticulate*, *Betta imbellis*, and *Trichopsis vittata*, *Euclionostomum* sp. have been found in the muscles [63, 64]. This digenean has been associated with disfiguration of fish, reduced growth rate and survival, loss of market value of both ornamental and food fish, compromised fish immunity leading to secondary infections by other pathogens, and mass death in juvenile fish when the intensity is high [63–65]. *Euclionostomum* sp. is considered to have zoonotic potential [66].

Gichohi et al. [22] found *Clinostomum* sp. (Figure 9) infecting the skin of O. *niloticus* in Upper River Tana basin in Kenya. *Clinostomum* sp. infections of the skin and muscles have also been reported by Maina et al. [17] in Kiambu County, Mavuti et al. [15] in Nyeri County, and Murugami et al. [18] and Waruiru et al. [19] in Kirinyaga County, Kenya. *Clinostomum* sp. was identified by its smooth body, oval‐shaped metacercariae, dorsoventrally flattened, and with a sucker around the anteroventral mouth and an additional sucker or acetabulum, and they appeared as yellowish grubs on the infected *O. niloticus* [15]. Studies in Kenya showed that the prevalence of *Clinostomum* sp. was high in earthen ponds because of the availability of numerous snail intermediate hosts [15, 17, 18].

Figure 9
*Clinostomum* sp. on *Oreochromis niloticus*, arrows showing yellow grubs on the scales (a) and isolated *Clinostomum* metacercaria (b) (source: Maina et al. [17]).

**Figure:**
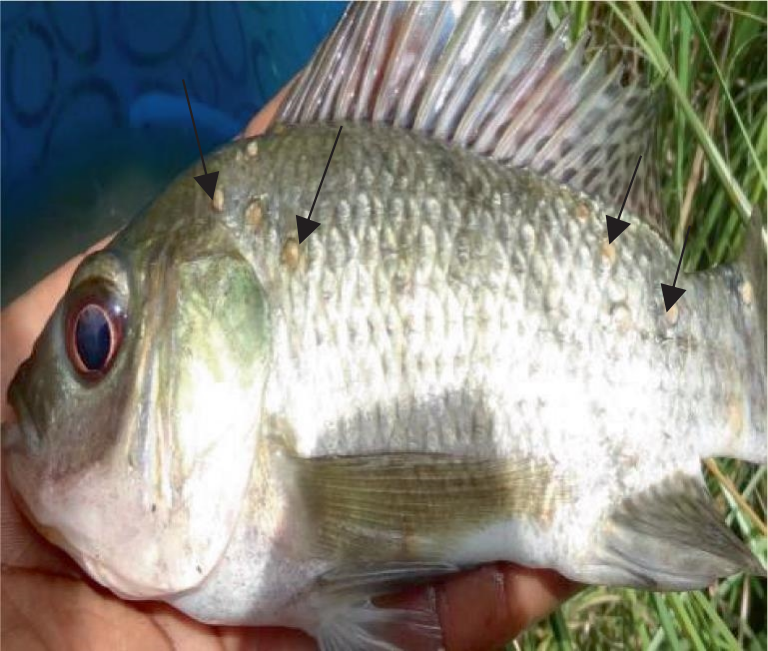
(a)

**Figure:**
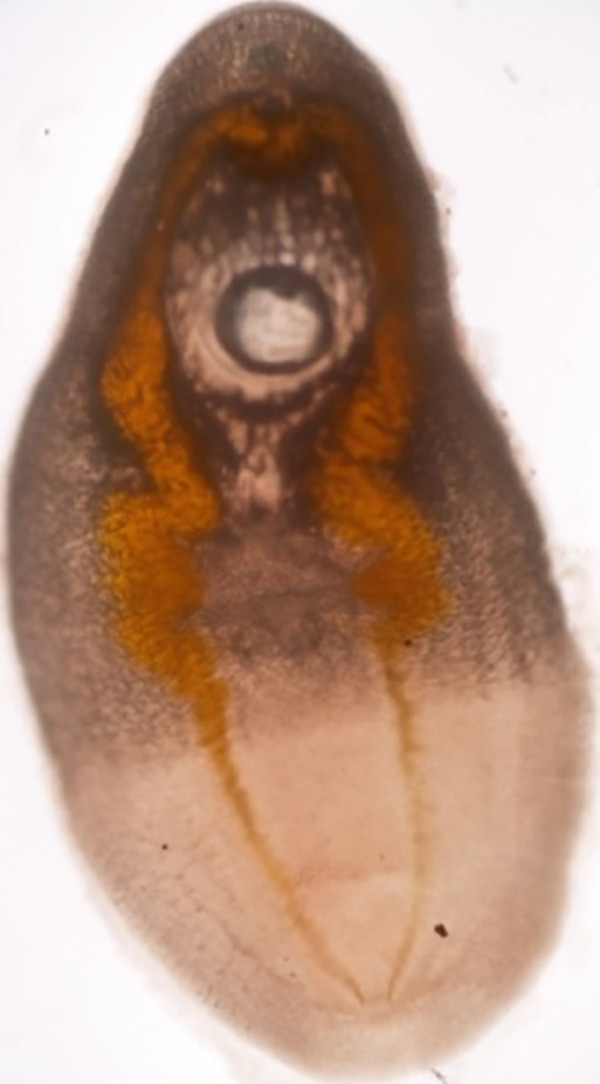
(b)

*O. niloticus* is considered an important cheap protein source in Kenya. Heavy clinostomiasis infections result in retarded growth, aberrant behavior, and mortality in fish hosts [67]. Fishermen and consumers frequently consider fish with accumulated yellow grubs unsuitable for human consumption, leading to rejection of the fish [68]. This trematode is considered to have zoonotic potential [69, 70].

### 5.3. Acanthocephalans

**Figure 10:**
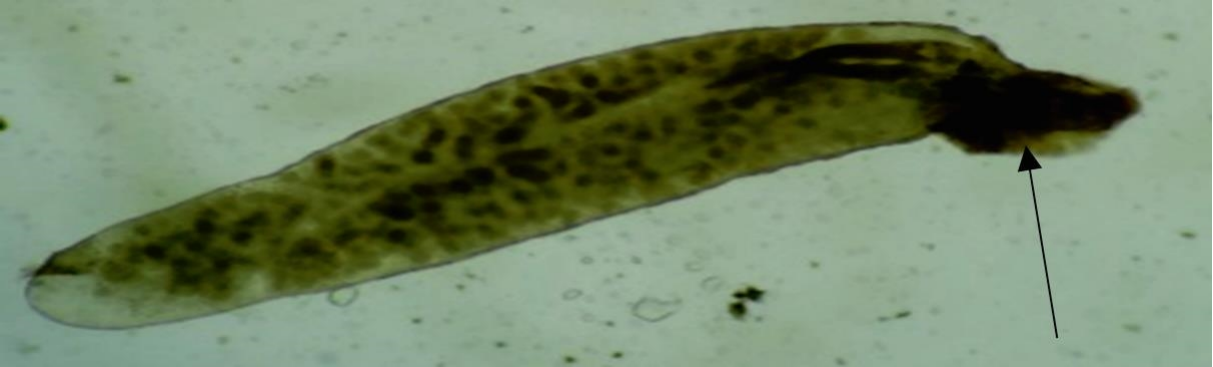
*Acanthocephalus* sp. with a protruding proboscis (arrow) was recovered from the intestines of *Oreochromis niloticus* in Taita Taveta County (source: Ageng′o et al. [41]).

*Acanthocephalus* sp. (Figure 10) have been recovered in the intestines of tilapia, catfish, and goldfish in Kenya [18, 19, 21]. The occurrence of *Acanthocephalus* sp. was attributed to piscivorous birds, which are the definitive hosts. Ponds located near water bodies where there were piscivorous birds had a higher prevalence of the parasite [18]. In Lake Naivasha, *P. kenyensis* has been recovered on *Oreochromis leucostictus* and *T. zillii* [35]. Acanthocephalans attach to the gastrointestinal mucosa of the host (mammals, birds, reptiles, amphibians, and fish) by use of a proboscis equipped with a series of hooks. Fish serve as definitive hosts to some acanthocephalans, which can affect their nutritive status due to absorption of nutrients in the gut, but they are not infective to humans. Other acanthocephalans, residing as larvae/juveniles in other compartments of the fish, use mammals like whales and seals as paratenic hosts. These are potentially zoonotic, as consumption of live larvae in infected fish may lead to human infection associated with severe abdominal symptoms [71].

Although some acanthocephalans have been associated with rare human infections elsewhere, those reported in Kenyan freshwater fish are predominantly of veterinary and ecological importance. Their zoonotic relevance remains speculative, particularly given the lack of evidence for human infection under local dietary practices.

### 5.4. Nematodes

**Figure 11:**
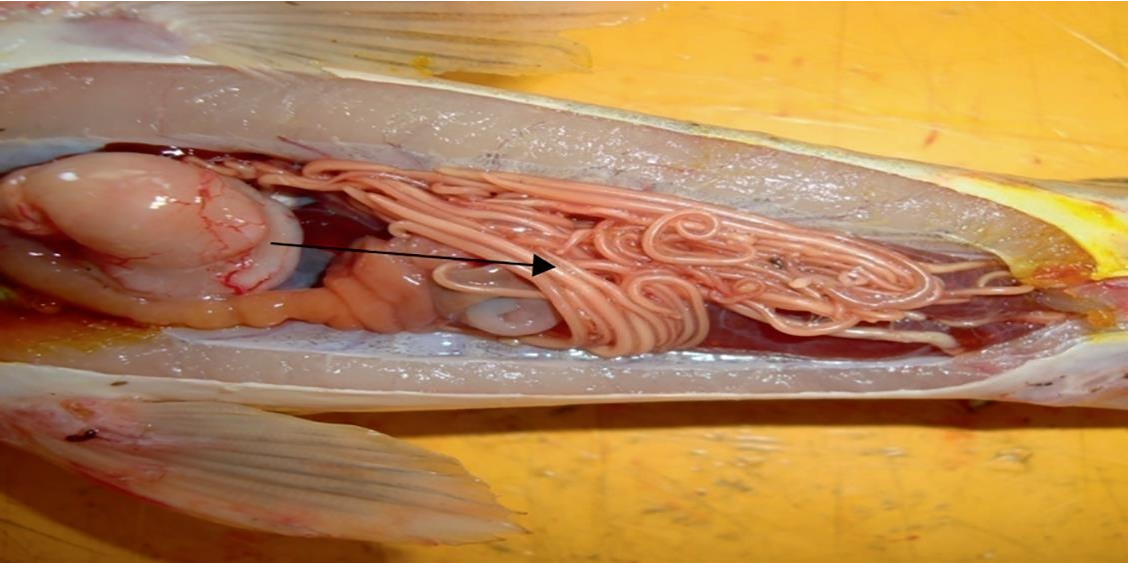
Third stage larvae (arrow) of *Contracaecum* sp. located in the abdominal cavity of a catfish (source: Mavutiet al. [15]).

*Contracaecum* sp. have been reported infecting the abdominal cavity of catfish and tilapia in Kenya [15, 18, 21, 22]. The prevalence of *Contracaecum* sp. (Figure 11) was high in areas where piscivorous birds prey on fish [18].

*Contracaecum* sp. are parasitic nematodes belonging to the Anisakidae family and have a global distribution and are of zoonotic significance [72]. Anisakidosis is a disease caused by infection with anisakid nematodes, including *Contracaecum* larvae in humans. Larval nematodes of the genus *Contracaecum*, belonging to the Anisakidae family, are of recognized zoonotic importance globally [72–74], where human anisakidosis has been linked to consumption of raw or undercooked fish [72]. In Kenya, however, evidence of human infection remains undocumented, and the public health relevance of *Contracaecum* currently rests on its potential risk rather than confirmed cases.

**Figure 12:**
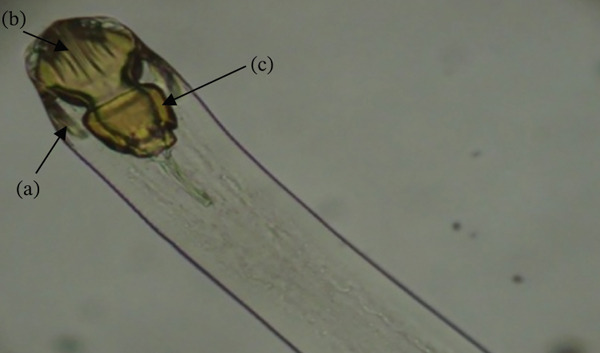
A *Paracamallanus* sp. from the gut of tilapia from Nyeri County showing the vertical chitinoid plates (a) and the buccal cavity divided into two levels: the upper smaller (b) and lower larger parts (c) (source: Mavuti et al. [15]).

*Camallanus* and *Paracamallus* (Figure 12) species have been recovered in the gastrointestinal tract of both catfish and tilapia in Kenya [15, 21, 22]. Mukwabi et al. [42] recovered *Procamallanus* and *Philometroides* species in aquaculture systems in Bungoma County infecting the gills and stomach of *O. niloticus*. These nematodes had a higher prevalence in earthen ponds due to the availability of intermediate hosts [21]. The authors also noticed that poor aquaculture husbandry practices like not treating or screening water from the river contributed to a high prevalence of *Camallanus* sp. infesting fish reared in plastic tanks. *Paracamallanus* sp. prevalence was high in liner ponds, especially the ones also using river water. There were chances that river water might have introduced feral fish infected with *Paracamallanus* sp. into the liner ponds [21]. The Camallanidae family is a globally distributed group of blood‐sucking parasitic nematodes that primarily infect marine and freshwater fish [75]. They are characterized by a well‐developed, usually orange‐colored buccal capsule and a life cycle involving a copepod intermediate host. *Clarias gariepinus* collected from Lake Heritage, Crocodile River in South Africa were found to harbor camallanid nematodes [76].

### 5.5. Cestodes

**Figure 13:**
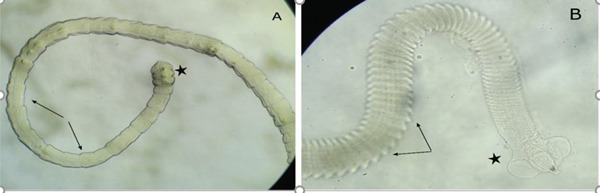
Pseudophyllidean and proteocephallid cestodes from catfish showing the scolex (star) and proglottids (arrows) (source: Murugami et al. [18]).

Fish cestode infections were reported by Gichohi et al. [22], Khamis et al. [77], and Murugami et al. [18] in Kirinyaga County, Kenya. Cestodes like the genus *Proteocephalus* (Figure 13b) have a uniform strobilar morphology and infect freshwater reptiles and fish [78]. Most cestodes are hermaphroditic; their proglottids contain both male and female organs and are capable of both self and cross fertilization [79]. The scolex, which is the anterior end, has attachment organs known as the bothria [65]. In a study involving the counties of Kirinyaga, Kisii, and Uasin Gishu in Kenya, Khamis et al. [77] found cestodes infecting *O. niloticus* and *C. gariepnus* in freshwater ponds, with catfish being the most infected.

*C. gariepinus* being omnivorous, they ingest a wide range of crustaceans such as copepods, which are intermediate hosts of cestodes [80]. Suckers on scolex destroy internal tissues of fish and these attachment sites provide entry point for secondary pathogens [81]. Cestode prevalence tends to be higher in piscivorous birds (definitive hosts) relative to the fish as recorded in Catfish where Caryophyllaeidea tapeworm along the shore of Masinga Dam [22]. *L. intestinalis* has been reported infecting *Barbus* sp. in Lake Baringo [36], and *A. macracantha* infects a wide range of fish including *Barbus* sp., *Oreochromis* sp. and *C. carpio* in different environments such as fish farms [31–33, 39, 47].

## 6. Predisposing Factors Associated With Fish Parasitism

The following section synthesises risk factors identified across multiple studies. A more quantitative summary of these factors, including odds ratios from specific studies, is presented in Tables 3 and 4 to illustrate the strength of these associations. A number of environmental and management‐related factors have been consistently linked to parasitic infections in Kenyan freshwater aquaculture systems.

**Table 3:** Odds ratio for fish infections against some farm practices in Kirinyaga County, Kenya.

Risk	X^2^ (*d* *f* = 1)	*p* < 0.05	Cramer′s V	Odds ratio
Use of livestock manure for pond fertilization	0.227^a^	0.634	0.064	1.500
Overstocked fishponds	0.094^a^	0.759	0.034	1.168
Feeding fish on homemade ration (livestock feeds and weed)	0.045^a^	0.832	0.024	1.128
Sharing fish nets and other equipment with other farmers	0.026^a^	0.873	0.017	1.088
Occurrence of floods	0.001^a^	0.978	0.003	1.018

*Note:* Superscipt letter (a) denotes insignificant associations (X2 (df = 1), P < 0.05). Source: Wanja et al. [6].

**Table 4:** Odds ratio for fish infections against some farm practices in Kericho and Bomet counties, Kenya.

Risk	X^2^ (*d* *f* = 1)	*p* < 0.05	Cramer′s V	Odds ratio
Ponds in valleys/gullies	0.368	0.544	0.329	2.028
Use of river water	0.058	0.809	0.405	1.654
Earthen ponds	0.448	0.503	0.322	2.023
Pond fertilization (livestock)	0.413	0.520	0.380	5.633

*Note:* (X2 (df = 1), p < 0.05); Source: Ageng′’o et al. [8].

### 6.1. Use of Livestock Manure

Livestock manure is widely used as pond fertilizer due to the high cost of commercial alternatives [17]. Studies in Kirinyaga County by Wanja et al. [20] and Kericho and Bomet by Ageng′o et al. [21] reported modest statistical associations between manure application and parasite prevalence (Kirinyaga: *χ* ^2^ = 0.227, *p* = 0.634, OR = 1.50; Table 3). The use of manure increases organic matter in ponds, which can deplete dissolved oxygen (DO) and stimulate microbial growth, weakening fish immunity and providing favorable conditions for parasites such as *Dactylogyrus*, *Gyrodactylus*, and *Diplostomum* species [6, 82]. These may contribute to selection, emergence, and spread of drug‐resistant pathogens, which pose serious threats to public health [83, 84]. Research has indicated that the use of antimicrobials as growth promoters in agriculture is associated with the emergence of antimicrobial‐resistant foodborne pathogens, which are relatively risky to human, animal, and environmental health [85, 86].

### 6.2. Mixed Stocking and Breeding Practices

Ponds containing mixed tilapia species or subject to uncontrolled breeding were more heavily infected with monogeneans (*Dactylogyrus* sp. and *Gyrodactylus* sp.) and ectoprotozoans, including *Trichodina*, *Ichthyobodo*, and *Epistylis species* [17, 21]. Overstocking increases direct contact among fish, enhancing parasite transmission. Statistical analysis indicated a low but measurable effect on infection risk (*χ* ^2^ = 0.094, *p* = 0.759, OR = 1.17; Table 3). Overstocking also contributes to water quality degradation, including reduced DO and increased ammonia, which further compromise fish health [6].

### 6.3. Feed Quality and Nutritional Stress

Limited access to high‐quality commercial feeds compels many farmers to use homemade rations (wheat bran, rice germ, or livestock feed), which are often protein deficient. According to Wanja et al. [6], fish fed on such diets had a 12.8% higher infection rate compared with those on commercial feeds. Odds ratio analysis confirmed a slight increase in infection risk (*χ* ^2^ = 0.045, *p* = 0.832, OR = 1.13). Poor nutrition impairs immunity, increasing susceptibility to parasites such as *Diplostomum* and *Clinostomum* species [87, 88].

### 6.4. Flooding and Environmental Stressors

Flooding in low‐lying ponds introduces organic debris, predators, and intermediate hosts (e.g., snails), promoting trematode infections (*Diplostomum sp.* and *Clinostomum sp.*) [6, 8, 89]. Although statistical associations in Kirinyaga were negligible (*χ* ^2^ = 0.001, *p* = 0.978, OR = 1.02; Table 3), field observations indicate transient increases in parasite loads following floods due to siltation and host proliferation.

### 6.5. Shared Farm Equipment

Reuse of harvest nets and other equipment without sanitation facilitates ectoparasite transmission, particularly *Trichodina* and *Epistylis* [17, 82, 90]. Odds ratio analysis (*χ* ^2^ = 0.026, *p* = 0.873, OR = 1.09) indicates a minor but measurable effect, especially when organic debris is present.

### 6.6. Pond Type and Design

Pond type strongly influences parasitic infections in aquaculture. Earthen ponds are consistently associated with higher parasite prevalence due to their structural and ecological characteristics. In Kericho and Bomet counties, Ageng′o et al. [8] reported that fish reared in earthen ponds had approximately twice the odds of infection compared with those in liner or concrete ponds (OR = 2.02, *χ* ^2^ = 0.448, *p* = 0.503; Table 4). High organic load, siltation, and debris accumulation in earthen ponds create favorable conditions for parasite proliferation, particularly helminths such as *Acanthocephalus*, *Contracaecum*, and *Camallanus* species [6, 90]. Earthen ponds often mimic natural aquatic systems, supporting aquatic vegetation and wildlife that act as reservoirs or intermediate hosts. For example, digenean trematodes such as *Diplostomum* and *Clinostomum* species thrive in ponds with abundant snails or mollusks [6, 90].

Interestingly, Ageng′o et al. [21] reported higher *Trichodina* sp. prevalence in liner ponds (11% of fish infected) compared with earthen ponds (2.1%), attributed to the limited capacity of liner ponds to dissipate nutrients. In contrast, earthen ponds benefit from horizontal seepage, which reduces organic accumulation and supports a more balanced microbial ecosystem. Despite this, earthen ponds still showed higher overall ectoparasite loads, with *Dactylogyrus* (8.8%) and *Acanthocephalus* (8.8%) species prevalence exceeding that of liner ponds (11.1% and 6%, respectively) and other systems such as plastic tanks, hatcheries, or concrete ponds [8, 21].

Across studies, poor water quality, high stocking density, and inadequate nutrition consistently emerged as key risk factors amplifying parasite outbreaks in all pond types [91]. Earthen ponds, being nutrient rich and prone to stagnation, particularly favor opportunistic protozoa and monogeneans, making regular monitoring, proper drainage, and biosecurity essential for effective parasite management.

### 6.7. Water Source Quality

Water source plays a critical role in determining parasite prevalence in aquaculture. The use of untreated river water is common among Kenyan fish farmers because it is abundant and inexpensive [17]. However, this practice significantly increases the risk of parasitic infections. Ageng′o et al. [8] reported that fish in river‐fed ponds had approximately twice the odds of infection compared with ponds using treated or alternative water sources (OR = 1.65, *χ* ^2^ = 0.058, *p* = 0.809; Table 4). Untreated river water often contains parasites originating from wild or feral fish, which can establish in farmed populations when no screening, filtration, or biosecurity measures are in place [92]. Parasites such as *Diplostomum*, *Contracaecum*, and *Acanthocephalus* species can be introduced via intermediate hosts like snails, crustaceans, or small fish, which thrive in river systems. The direct connection to natural water bodies bypasses any environmental or mechanical barriers that would otherwise limit parasite entry, making river‐fed ponds particularly vulnerable to outbreaks. Effective water management, including filtration, sedimentation, or partial treatment, is therefore essential to reduce exposure to parasites and maintain fish health in these systems.

### 6.8. Pond Location and Biosecurity

Ponds located in valley bottoms or open‐access areas show higher infection rates due to increased runoff, wildlife access, and inadequate fencing. Odds ratio analysis indicated that valley ponds were twice as likely to harbor parasites (OR = 2.03, *χ* ^2^ = 0.368, *p* = 0.544; Table 4) [7]. Poor biosecurity facilitates parasite introduction and spread, highlighting the need for fencing, monitoring, and controlled access.

## 7. Parasite Genera in Kenyan Fish and Their Predisposing Factors

### 7.1. *Contracaecum* Species

Nematodes of the genus *Contracaecum* are some of the most common parasites found in Kenyan lakes, especially Lake Naivasha. Otachi et al. [33] showed that *C. multipapillatum* frequently infects *O. leucostictus*, a tilapia species that feeds in areas rich in aquatic invertebrates. These infections are strongly influenced by the presence of fish‐eating birds, which serve as the parasite′s final hosts. The risk increases in polluted or nutrient‐rich environments, where both bird populations and intermediate hosts thrive. Pollution also weakens fish immunity, making them more likely to succumb to nematode infections, as highlighted by Otachi et al. [32] and Plessl et al. [93] (Table 5).

**Table 5:** Linkages between fish parasite genera, hosts, and predisposing factors in Kenyan freshwater systems.

Parasite genera	Common host(s)	Key risk factors/predisposing factors	References
*Diphyllobothrium*	*Tilapia zillii*; *Oreochromis niloticus*; *Clarias gariepinus*	Consumption of intermediate hosts (copepods, small fish); poor water sanitation; high stocking density	Aloo [35]; Khamis et al. [65]
*Schyzocotyle/Bothriocephalus*	*T. zillii*; *Oreochromis leucostictus*	Eutrophic waters; presence of definitive hosts (birds); overcrowding; low water flow	Aloo [35]
*Contracaecum*	*C. gariepinus*; *O. niloticus*	Ingestion of infected crustaceans; open water systems; poor fish handling	Aloo [35]; Murugami et al. [17, 27]; Ageng′o et al. [8, 21]
*Clinostomum*	*T. zillii*; *O. leucostictus*	Stagnant or slow‐flowing waters; presence of piscivorous birds; seasonal temperature changes	Waruiru et al. [19]; Otachi et al. [32, 33]
*Trichodina*	*T. zillii*; *O. niloticus*	High stocking density; poor water quality (i.e., low dissolved oxygen; handling stress	Ojwala et al. [31]; Adamba et al. [37]; Otachi et al. [32, 33]; Ageng′o et al. [8, 21]
*Gyrodactylus*	*C. gariepinus*; *T. zillii*	High density culture; rapid temperature fluctuations; poor hygiene; mechanical injuries	Ojwala et al. [31]; Adamba et al. [37]; Otachi et al. [32]; Ageng′o et al. [8, 21]
*Dactylogyrus*	*T. zillii*; *O. leucostictus*; *O. niloticus*; *C. carpio*	Poor water quality; overcrowding; introduction of infected fingerlings	Ojwala et al. [31]; Adamba et al. [37]; Otachi et al. [32]
*Lernaea*	*O. niloticus*; *C. gariepinus*	Poor water hygiene stagnant ponds; high host density; seasonal temperature peaks	Ojwala et al. [31]; Aloo [35]
*Camallanus*; *Procamallanus*; *Paracamallanus*; *Philometroides*	*C. gariepinus*; *T. zillii*	Infection via intermediate hosts (copepods); open water systems; inadequate pond management	Aloo [35]; Mukwabi et al. [42]; Mavuti et al. [15]

### 7.2. *Ligula* Species

The tapeworm *L. intestinalis* is known for its dramatic effects on fish behavior. Britton et al. [36] documented cases where infected fish swam abnormally and became easy prey for birds, the parasite′s definitive hosts. What predisposes fish to *Ligula* infections is the combination of shallow vegetated habitats, abundant copepods (the first intermediate hosts), and access by piscivorous birds. Lakes that experience eutrophication or habitat disturbance tend to offer ideal conditions for the parasite′s complicated life cycle to thrive.

Apart from *Ligula*, several other cestodes parasitize tilapia and related species. Aloo [35] reported a notable presence of these tapeworms in *T. zillii* and *O. leucostictus*, linking infections to the fish′s benthic feeding habits and regular contact with infected copepods. Shallow, nutrient‐enriched waters with abundant zooplankton significantly increase the odds of exposure. Human‐driven changes such as increased turbidity and runoff into lakes tend to support the intermediate hosts that cestodes rely on (Table 5).

### 7.3. Digenean Trematodes

These (*Diplostomum*, *Clinostomum*, *Euclionostomum*, and *Neascus*) are widespread in Kenyan tilapia populations. Shigoley et al. [28] described striking anatomical details of *Clinostomum cutaneum* metacercariae in *O. niloticus*, bringing attention to how these parasites embed themselves in fish tissues. Their transmission depends heavily on snail intermediate hosts. Warm, shallow, plant‐rich waters common near shorelines create the perfect setting for snail populations and cercariae to flourish. In aquaculture, trematode infections become more frequent when ponds have poor drainage, stagnant water, or excessive vegetation [31, 42]. Several digenean trematodes identified in Kenyan fish, including *Clinostomum* and *Euclinostomum* species, have been implicated in zoonotic infections in other regions. Within Kenya, their importance is primarily economic and aesthetic, as infections often lead to rejection of fish at harvest and markets. Any zoonotic significance should therefore be regarded as theoretical in the absence of documented human cases.

### 7.4. Monogeneans

Monogeneans, particularly species of *Cichlidogyrus*, are some of the most problematic gill parasites in tilapia. Rindoria et al. [34] found high infections of these parasites in *O. niloticus* and *O. leucostictus* from Lake Naivasha. Monogeneans reproduce quickly in warm, shallow waters exactly the conditions found in many littoral zones. In fish farms, the issue often gets worse because of poor water quality, high stocking densities, and infrequent pond cleaning [42]. Stressful conditions such as low oxygen or high ammonia [31, 42] give monogeneans an even greater advantage.

### 7.5. Endohelminths of *C. gariepinus*

The North African catfish, *C. gariepinus*, is known to carry a wide range of helminths. Maraganga et al. [94] documented extensive parasite diversity in populations from Lakes Naivasha and Ol′Bolossat. Because this species feeds on the bottom and consumes a variety of prey, it is more likely to ingest infective stages of many parasites. Environmental disturbances such as flooding in Lake Baringo, for instance, also disperse intermediate hosts and expand parasite habitats, as observed by Kiprono [95]. These events often translate to increased parasite transmission.

### 7.6. Parasites in Introduced Species

Introduced fish species such as Nile perch and Nile tilapia often bring ecological ripple effects, and parasites are no exception. Outa et al. [39] showed that both Nile perch (*L. niloticus*) and farmed Nile tilapia (*O. niloticus*) in Lake Victoria host diverse parasite communities. Because these species spread widely and interact with many ecological niches, they create new pathways for parasite exchange between native species and newcomers. Habitat modification, fishing pressure, and ecological imbalance can magnify these interactions, leading to shifts in parasite transmission across the ecosystem. Otachi et al. [33] observed that the occurrence of *D. minutus* and *D. extensus* in Lake Naivasha was as a result of the invasion of the lake by *C. carpio* (Table 5).

### 7.7. Parasites of *Oreochromis esculentus* and *Protopterus aethiopicus*

In Lake Kanyaboli, Olonde [96] explored the parasite fauna of the threatened *O. esculentus* and the African lungfish (*P. aethiopicus*). Using both molecular and morphological methods, the study revealed complex parasite communities influenced by seasonal flooding, shared feeding grounds, and agricultural runoff entering the lake. These conditions increase contact between fish and the intermediate hosts required for parasite life cycles (Table 5).

### 7.8. Overall Parasite Richness and Effects on Productivity

A broader synthesis by Kibet et al. [47] emphasized that parasitism is strongly tied to environmental and human pressures across Kenyan inland waters. Their review showed that polluted, fragmented, or heavily exploited lakes tend to have higher parasite loads. In aquaculture, productivity can drop sharply when ponds are poorly managed, overcrowded, or set in environments with contaminated water sources (Table 5).

### 7.9. Mixed Parasite Communities in Aquaculture

Farmed fish often carry a mixture of ectoparasites (monogeneans, protozoans, and crustaceans) and endoparasites such as nematodes, trematodes, cestodes, and acanthocephalans. Mukwabi et al. [42] reported varied parasite assemblages infecting *O. niloticus* across Kenyan farms. The risk is especially high where stocking densities are excessive and water exchange is limited. Ojwala et al. [31] further showed that parasites flourish when ponds have poor water quality indicators such as high ammonia and low oxygen because stressed fish have less ability to fight infections. Table 5, shows linkage between fish parasite genera and host in varying culture systems.

### 7.10. Coinfections of Parasites With Other Pathogens

Parasitic infections in freshwater fish often do not occur in isolation. Instead, they frequently open the door for secondary infections, compounding their impact on fish health and survival. For instance, ciliate ectoprotozoans such as *Trichodina* and *Epistylis* sp. are known to cause mechanical damage to fish skin and fins. *Epistylis* attaches directly to the epidermis, causing superficial wounds, whereas the rapid, circular movement of *Trichodina*, aided by its lateral hooks, creates abrasions that compromise the protective mucus and epithelial layers [50, 97].

In a study conducted in Kirinyaga County, Wanja et al. [20] documented naturally occurring coinfections in tilapia involving black spot disease caused by *Neascus* sp. alongside multiple bacterial infections. *Aeromonas* sp., *Enterobacter cloacae*, *Klebsiella pneumoniae*, and *Micrococcus luteus* were the most prevalent isolates. These findings highlight how parasite‐induced stress and tissue damage can predispose fish to bacterial, viral, and fungal coinfections, escalating both morbidity and mortality in affected populations [98].

### 7.11. Role of Piscivorous Birds in Spread of Fish Parasites

Piscivorous birds are a growing concern for fish farmers in Kenya due to their role in the transmission of parasitic infections. As definitive hosts, these birds shed parasite eggs or larvae that infect fish serving as intermediate hosts [34]. The resulting infections can stunt fish growth, increase mortality rates, and compromise market quality, making infected fish less visually appealing and often rejected by consumers [61, 62]. Evidence from Kirinyaga County highlights this dynamic. Murugami et al. [18] reported the highest prevalence of parasitic infections, 69% in Kirinyaga West and 68% in Mwea East subcounties compared with lower rates in Kirinyaga Central Subcounty. These areas host major fish farms such as the Sagana Aquaculture Research Centre and Mwea Aqua Fish Farm, both of which attract large populations of piscivorous birds. Species frequently associated with parasite transmission include cormorants, kingfishers, herons, spoonbills, and hamerkops. These birds were found to harbor a range of parasites, including *Pseudophyllidean* and *Proteocephalid* cestodes, as well as members of the genera *Acanthocephalus*, *Contracaecum*, and *Clinostomum* [18].

### 7.12. Influence of Water Quality on Fish Parasitism

Water quality plays a critical role in shaping fish health and their susceptibility to parasites. Fish live in constant interaction with waterborne pathogens, including bacteria, viruses, fungi, and parasites, and suboptimal water conditions can impair immune function, increasing vulnerability to infection [99].

Poor water quality is particularly stressful and has been linked to outbreaks of diseases such as viral hemorrhagic septicemia [100]. In Kenyan aquaculture systems, Waruiru et al. [19] found that high levels of phosphate and ammonia nitrogen correlated positively with parasite infections (Table 6). These nutrients often drive eutrophication, which in turn promotes algal blooms. As these blooms decay, they deplete oxygen levels in the water, creating hypoxic or anoxic conditions that weaken fish and promote parasite proliferation [101].

**Table 6:** Correlation coefficient of overall fish parasite prevalence in Kirinyaga County, Kenya.

Water parameter	Correlation coefficient	Nature of associations
Temperature	−0.139	Negligible
Dissolved oxygen	−0.406	Low negative
Ph	−0.343	Low negative
Ammonia‐free nitrogen	0.582	Moderate positive
Nitrates	0.286718	Low positive
Nitrites	−0.0633	Negligible
Phosphates	0.412486	Low positive

*Note:* Source: Waruiru et al. [19].

Waruiru et al. [19] showed that the abundance of *C. cutaneum* was positively related to DO and pH (Table 7).

**Table 7:** Correlation coefficient of parasite abundance against water quality parameters in Kirinyaga County, Kenya.

Physicochemical parameters of water				
Parasite genera	Temperature	Dissolved oxygen	Ph	Ammonia‐free nitrogen
*Diplostomum*	−0.2	−0.4	−0.3	−0.1
*Clinostomum cutaneum*	0.4	0.5∗	0.5∗	−0.1
*Neascus*	0.2	−0.05	0.22	−0.06
*Gyrodactylus*	0.2	0.2	0.3	−0.1
*Dactylogyrus*	0.02	0.03	0.07	−0.2
*Acanthocephalus*	0.05	0.04	0.17	−0.1
*Piscicola* leeches	0.2	0.2	0.3	−0.1
Pseudophyllid cestodes	0.2	0.3	0.2	−0.1

*Note:* Source: Waruiru et al. [19].

Asterisk denotes that *Clinostomum cutaneum* was positively correlated with dissolved oxygen and PH.

Ageng′o et al. [21] explored how water quality parameters shape the diversity and prevalence of fish parasites in *O. niloticus* across Kericho, Bomet, and Nakuru counties (Table 8). Their findings revealed that several parasite groups are strongly influenced by the chemical and physical conditions of freshwater environments. Notably, the prevalence of *Diplostomum* sp. was positively associated with elevated water temperature, pH, and sulfate levels. These conditions promote aquatic plant growth, which in turn supports snail populations, key intermediate hosts in the *Diplostomum* life cycle [37]. Similarly, electrical conductivity and pH were linked to higher occurrences of *Acanthocephalus* and *Euclinostomum* species.

**Table 8:** Influence of water quality parameters on occurrence of fish parasites in Kericho, Bomet, and Nakuru counties, Kenya.

Parasite Genera	Nitrites (mgL^−1^)	Nitrates (mgL^−1^)	CaCo^3^ (mgL^−1^)	Sulfates (mgL^−1^)	DO (mgL^−1^)	Temp (°C)	pH	PO_4_ (mgL^−1^)	Electrical conductivity (*μ*S/cm)	Turbidity (NTU)
*Diplostomum*	**−**1.00	0.15	**−**1.00	1.00	**−**0.80	0.87	0.66	**−**0.08	0.40	**−**0.87
*Acanthocephalus*	—	**−**0.82	—	—	**−**0.89	**−**0.05	0.97	**−**0.93	1.00	0.06
*Dactylogyrus*	**−**1.00	0.91	**−**1.00	1.00	0.07	0.88	**−**0.28	0.79	**−**0.56	**−**0.89
*Euclinostomum*	—	‐0.82	—	—	‐0.89	**−**0.05	0.97	**−**0.93	1.00	0.06
*Riboscyphidia*	**−**1.00	0.95	**−**1.00	1.00	0.17	0.83	**−**0.37	0.85	**−**0.63	**−**0.84
*Epistyis*	**−**1.00	0.94	**−**1.00	1.00	0.15	0.84	**−**0.36	0.84	**−**0.62	**−**0.85
*Trichodina*	**−**1.00	**−**0.80	**−**1.00	1.00	**−**0.91	**−**0.01	0.98	**−**0.92	1.00	0.03
*Contracaecum*	**−**1.00	0.40	**−**1.00	1.00	**−**0.61	0.97	0.43	0.18	0.14	**−**0.97
*Paracamallanus*	**−**1.00	0.96	**−**1.00	1.00	0.22	0.80	**−**0.42	0.87	**−**0.67	**−**0.81
*Camallanus*	**−**1.00	0.90	**−**1.00	1.00	0.05	0.89	**−**0.26	0.78	**−**0.54	**−**0.89

*Note:* Source: Ageng′o et al. [21].

Abbreviations: (*μ*S/cm), microsiemens per centimeter; CaCO^3^, calciumcarbonate; DO, dissolved oxygen; mgL^−1^, milligrams per liter; NTU, nephelometric turbidity units; PO_4_, phosphates; Temp, temperature.

The prevalence of ciliated ectoprotozoans like *Epistylis* and *Riboscyphidia* increased with rising levels of phosphates, sulfates, nitrates, and warmer temperatures. This aligns with observations by Pádua et al. [102], who noted that nutrient enrichment in aquatic systems encourages ciliate proliferation. *Trichodina* sp. also showed positive correlations with electrical conductivity, pH, and sulfate concentrations, reinforcing earlier findings by Adamba et al. [37].

Furthermore, nematodes such as *Paracamallanus*, *Camallanus*, and *Contracaecum* species were more prevalent in waters with higher temperatures and increased nitrate, phosphate, and sulfate concentrations. These conditions enhance the mobility and availability of infective larvae, increasing transmission opportunities to susceptible fish hosts [37].

## 8. Conclusions and Recommendations

This review highlights the considerable burden and diversity of parasitic infections affecting freshwater fish in Kenya. Across the various studies examined, a total of 45 parasite genera were identified, with the highest diversity observed in *Oreochromis* species, which hosted up to 37 parasite types. *C. gariepinus* followed, harboring 11, *Barbus* sp. 10, *Haplochromis* sp. 8, *C. carpio* 6, and *L. niloticus* 4, whereas ornamental fish were infected by three genera. From the review, the invasion of Lake Victoria by *L. niloticus* led to the establishment of *H. ghaffari*, *D. lacustris*, and *Cucullanus*, whereas the invasion of Lake Naivasha by *C. carpio* led to the establishment of *D. minutus*, *D. extensus*, and *Acolpenteron* sp. These newly established parasites are likely to enlarge their territory by spilling over to endemic fish species within the lakes.

The parasites identified span a wide range of taxa, including 6 nematodes, 7 digeneans, 6 monogeneans, 5 crustaceans, 3 acanthocephalans, 6 cestodes, and 12 protozoans. High diversity of helminths, particularly in wild and poorly managed aquaculture systems, was a result of the ecological adaptability of these parasites and their potential impact on fish health and productivity.

Hatchery‐raised *Oreochromis* species were most affected by ectoparasites, with *Gyrodactylus* sp. emerging as the most prevalent, highlighting both the vulnerability of juvenile fish and the biosecurity challenges in hatchery environments.

Overall, the zoonotic relevance of fish parasites reported in Kenya should be interpreted with caution. Although several taxa identified in freshwater fish are recognized as zoonotic agents globally, there is insufficient evidence linking these parasites to human disease in Kenya. Their primary significance lies in their impact on fish health, aquaculture productivity, and market acceptability. Nevertheless, changing consumption patterns, informal fish processing, and increasing aquaculture intensification justify continued surveillance and public awareness as precautionary public health measures.

Several key risk factors were consistently associated with parasitic infections across studies. Poor water quality characterized by low DO, high ammonia, and eutrophication was a prominent contributor. Risk was also elevated in systems where nets and equipment were shared, ponds were overstocked, or postharvest pond sediments were not cleared before restocking.

Given these findings, a number of targeted recommendations emerged as follows:

Modern diagnostic tools, particularly molecular techniques such as PCR and next‐generation sequencing (NGS), should be adopted for accurate identification and monitoring of fish parasites. These methods can help distinguish cryptic species and detect coinfections that are often missed with traditional microscopy.

There is a critical need to support research and innovation focused on developing vaccines or immunostimulants targeting economically and zoonotically important parasites. Collaborative programs involving government agencies, universities, and the private sector should be prioritized.

Public health education campaigns should be expanded to raise awareness of the zoonotic risks associated with consuming raw or undercooked fish, especially in high‐risk regions or where traditional consumption habits persist.

The use of livestock manure in aquaculture should be regulated. Farmers should be encouraged to adopt ecofriendly alternatives such as biofertilizers and waste‐to‐energy technologies, which reduce pathogen load while contributing to circular economy goals.

Fish nutrition remains a cornerstone of disease resistance. Promoting balanced, sustainable feeds that enhance fish immunity can help reduce susceptibility to parasitic infections.

Finally, building capacity through continuous training of extension workers and farmers is essential. Good aquaculture husbandry practices, especially those that promote biosecurity, pond hygiene, and water quality management, must be institutionalized through farmer field schools, demonstration farms, and digital extension platforms.

## Funding

No funding was received for this manuscript.

## Conflicts of Interest

The authors declare no conflicts of interest.

## Data Availability

This is a review article, and no primary data were generated. Any data analyzed are within this article and its supporting information files.
